# Table of Meteorological Conditions in the City of Buffalo for October, 1854

**Published:** 1854-12

**Authors:** Sanford B. Hunt


### ART. XI.—*Record of Meteorological Conditions at Buffalo, for October,* 1854. By SANFORD B. HUNT, M. D.

| Date. | DAILY. In. of Barom. | DAILY. In. of Rain. | UPPER STATION. At 7, A. M. Am't of Clouds | At 7, A. M. Force and Course of Wind. | At 7, A. M. Hygrometer. Temp'ture. | At 7, A. M. Hygrometer. Dew Point. | At 7, A. M. Hygrometer. Humidity. | UPPER STATION. 2, P. M. Am't of Clouds | 2, P. M. Force and Course of Wind. | 2, P. M. Hygrometer. Temp'ture. | 2, P. M. Hygrometer. Dew Point. | 2, P. M. Hygrometer. Humidity. | LOWER STAT'N. 2, P. M. Hygrometer. Temp'ture. | LOWER STAT'N. 2, P. M. Hygrometer. Dew Point. | LOWER STAT'N. 2, P. M. Hygrometer. Humidity. | UPPER STATION. 9, P. M. Am't of Clouds | 9, P. M. Force and Course of Wind. | 9, P. M. Hygrometer. Temp'ture. | 9, P. M. Hygrometer. Dew Point. | 9, P. M. Hygrometer. Humidity. | DAILY. Mean Temperature. | DAILY. Mean Dew Point. | DAILY. Mean Humidity. | Greatest range of Temperature. |
|---|---|---|---|---|---|---|---|---|---|---|---|---|---|---|---|---|---|---|---|---|---|---|---|---|
| 1 | | .116 | 10 | 1. S.W. | 60. | 48.33 | 655 | 10 | 2. S. | 61. | 56.33 | 860 | 65. | 53.33 | 696 | 2 | 3. W. | 62. | 52.66 | 760 | 61. | 52.44 | 758 | 2 |
| 2 | 29.0 | | 0 | 0 | 47. | 44.66 | 760 | 0 | 1. S.W. | 60. | 50.66 | 760 | 66. | 54.33 | 696 | 8 | 1. S,W. | 56. | 49. | 810 | 54.3 | 48.05 | 777 | 13 |
| 3 | 28.3 | .240 | 10 | 1. S. | 54. | 49.33 | 860 | 10 | 3. S. | 67. | 60. | 810 | 69. | 59.66 | 760 | 10 | 6. S.E. | 61. | 51.66 | 760 | 60.6 | 53.6 | 776 | 13 |
| 4 | 29.2 | .020 | 10 | 4. W. | 51. | 41.66 | 760 | 10 | 4. W. | 55. | 43.33 | 696 | 60. | 50.33 | 760 | 1 | 1. W. | 47. | 40. | 809 | 51. | 41.6 | 776 | 8 |
| 5 | 29.5 | | 0 | 1. W. | 43. | 39.33 | 860 | 4 | 1. S. | 55. | 41. | 696 | 65. | 51. | 656 | 0 | 1. S. | 47. | 37.66 | 760 | 48.3 | 39.3 | 759 | 12 |
| 6 | 29.3 | | 0 | 1. S. | 52. | 40.33 | 689 | 0 | 1. S.W. | 68. | 49.33 | 560 | 67. | 60. | 809 | 0 | 1. S.W. | 62. | 45.33 | 620 | 60.6 | 45. | 623 | 16 |
| 7 | 29.4 | | 0 | 3. S.W. | 67. | 50.33 | 609 | 0 | 3. S.W. | 70. | 51.33 | 569 | 70. | 58.33 | 696 | 0 | 1. S.W. | 62. | 57.33 | 860 | 66.6 | 53. | 676 | 8 |
| 8 | | | 1 | 1. S.W. | 64. | 57. | 809 | 0 | 1. S.W. | 71. | 57. | 656 | | | | 5 | 2. S.W. | 64. | 54.66 | 763 | 66.6 | 56.5 | 743 | 7 |
| 9 | 29.2 | | 0 | 2. S. | 62, | 55. | 809 | 3 | 1. S.W. | 68. | 58.66 | 760 | 67. | 55.33 | 696 | 10 | 2. W. | 62. | 57.33 | 860 | 64. | 53. | 809 | 6 |
| 10 | 29.2 | .300 | 8 | 2. N.E. | 56. | 51.33 | 809 | 2 | 2. N. | 60. | 55.33 | 860 | 71. | 59.33 | 696 | 0 | 0 | 52. | 49.66 | 939 | 56. | 52. | 869 | 8 |
| 11 | 29.1 | | 0 | 1. S. | 59. | 52. | 809 | 3 | 1. S.W. | 68. | 56.33 | 696 | 69. | 59.66 | 763 | 0 | 0 | 63. | 56. | 809 | 65.3 | 54.7 | 771 | 9 |
| 12 | 28.8 | .550 | 10 | 2. S. | 64. | 57. | 809 | 10 | 0 | 64. | 61.66 | 939 | 78. | 68.66 | 763 | | | | | | 64. | 59.3 | 874 | 0 |
| 13 | 29.0 | | 5 | 1. E. | 53. | 48.66 | 860 | 6 | 1. N.E. | 59. | 49.66 | 760 | 68. | 58.66 | 763 | 0 | 1. E. | 48. | 45.66 | 939 | 53.3 | 47.9 | 853 | 11 |
| 14 | 28.8 | .312 | 10 | 1. S.W. | 53. | 48.33 | 860 | 10 | 3. N.W. | 54. | 49.33 | 860 | 68. | 61. | 809 | 2 | 3. W. | 45. | 38. | 809 | 50.6 | 45.2 | 843 | 9 |
| 15 | | | 10 | 3. N. | 40. | 35.33 | 860 | 2 | 3. N.W. | 47. | 33. | 656 | 62. | 52.66 | 763 | | | | | | 43.5 | 34.2 | 758 | 7 |
| 16 | 29.0 | | 10 | 1. N.W. | 43. | 38.33 | 860 | 10 | 2. N.W. | 51. | 41.6 | 750 | 64. | 54.66 | 760 | 10 | 1. N.W. | 48. | 41. | 809 | 47.3 | 40.3 | 809 | 8 |
| 17 | 29.0 | .167 | 9 | 1. N.W. | 46. | 36.66 | 860 | 8 | 1. N.W. | 51. | 37. | 656 | 60. | 48.33 | 697 | | | | | | 48.5 | 36.8 | 758 | 5 |
| 18 | 29.0 | | 3 | 3. W. | 42. | 35. | 809 | 5 | 2. W. | 45. | 35.66 | 760 | 52. | 38. | 656 | 10 | 1. W. | 40. | 35.33 | 860 | 44. | 34.6 | 809 | 3 |
| 19 | 29.8 | | 10 | 1. W. | 35. | 30.33 | 860 | 5 | 1. W. | 44. | 32.33 | 696 | 61. | 30.66 | 367 | 5 | 1. W. | 36. | 31.33 | 860 | 38.3 | 30.3 | 805 | 9 |
| 20 | 29.3 | | 10 | 1. W. | 35. | 30.33 | 860 | 5 | 1. S.E. | 52. | 35.66 | 620 | 62. | 45.33 | 620 | 10 | 1, S.E. | 45. | 40.33 | 860 | 44. | 35.4 | 780 | 17 |
| 21 | 29.5 | | 0 | 0 | 36. | 33.66 | 939 | 0 | 1. N.W. | 59. | 40.33 | 560 | 65. | 46.33 | 558 | 0 | 1. N.W. | 48. | 43.33 | 860 | 47.6 | 39.1 | 786 | 23 |
| 22 | | .085 | 5 | 1. N.W. | 42. | 37.33 | 860 | 10 | 0 | 52. | 42.66 | 760 | 66. | 54.33 | 699 | 10 | 0 | 50. | 45.33 | 860 | 48. | 41.4 | 823 | 10 |
| 23 | 29.3 | | 10 | 1. S.E. | 50. | 40.66 | 763 | 10 | 1. S.E. | 56. | 42.33 | 696 | 58. | 46.33 | 696 | 10 | 0 | 54. | 45.66 | 809 | 53.3 | 42.7 | 756 | 6 |
| 24 | 29.2 | | 9 | 1. S.E. | 54. | 44 66 | 763 | 0 | 1. S.W. | 64. | 50. | 656 | 66. | 49.33 | 620 | 0 | 0 | 52. | 47.33 | 860 | 56.6 | 47.3 | 759 | 12 |
| 25 | 29.5 | | Fog | 0 | 50. | 47.66 | 939 | 0 | 1. S. | 60. | 53. | 809 | 64. | 52.33 | 699 | 0 | 0 | 54. | 49.33 | 860 | 54.6 | 49.9 | 869 | 10 |
| 26 | 29.2 | | 0 | 0 | 45. | 42.66 | 939 | 0 | 1. S. | 65. | 51. | 656 | 68. | 61. | 809 | 0 | 1. S. | 56. | 49. | 809 | 55.3 | 47.5 | 798 | 20 |
| 27 | 29.3 | | 0 | 1. S. | 47. | 44.66 | 939 | 2 | 1. S. | 65. | 51. | 656 | 75. | 58.3 | 620 | 0 | 0 | 57. | 52.33 | 860 | 56.3 | 49.3 | 818 | 18 |
| 28 | 29.4 | | 8 | 1. S. | 49. | 46.66 | 939 | 4 | 1. S. | 66. | 52. | 656 | 76. | 53.8 | 530 | 4 | 1. S. | 58. | 53.33 | 860 | 57.7 | 50.6 | 818 | 17 |
| 29 | | .103 | 10 | 1. S. | 61. | 56.33 | 860 | 10 | 1. S. | 67. | 60. | 809 | 74. | 60. | 656 | 10 | 1. S. | 65. | 60.33 | 860 | 62.3 | 58.8 | 843 | 6 |
| 30 | 29.2 | | 10 | 1. S. | 57. | 54.66 | 939 | 9 | 0 | 69. | 57.33 | 696 | 71. | 61.66 | 763 | 3 | 0 | 64. | 59.33 | 860 | 60. | 57.1 | 832 | 12 |
| 31 | 28.8 | .117 | 10 | 3. S·W. | 60. | 57.56 | 939 | 9 | 3. S.W. | 62. | 55. | 809 | 72. | 53.33 | 560 | 10 | 4. S.W. | 58. | 55.66 | 939 | 60. | 56.1 | 896 | 4 |
| | | 2.010 | | | 50.87 | 45.03 | 832 | | | 59.84 | 48.71 | 721 | 66.63 | 57.19 | 688 | | | 54.14 | 48.35 | 833 | 54.8 | 47.8 | 794 | 9.97 |

The meteorological observations for October, have assumed a more extended character than I have hitherto been able to give them. Only two or three additions are now wanting to make them perfect, and the labor they involve has become so habitual as to be unnoticed.

*Temperature.*—It will be seen that the extreme range of temperature upon any day was upon the 21st, when there was a variation of 23 degrees between 6, A. M. and 2, P. M. The minimum range was 2°, upon the 1st of the month. The average temperature at 7, A. M., was 50° 87′; at 2, P. M., 59° 84′; at 9, P. M., 54° 80′. Thus there was an average difference between 7, A. M. and 2, P. M., of 8° 97′; and between 2, P. M. and 9, P. M., of 5° 04′. The dew point was more permanent, standing at 45° 03′, at 7, A. M., at 58° 71′, at 2, P. M., and at 47° 80′, at 9, P. M. These constitute high points of maximum density for this season and latitude. The relative humidity was correspondingly high, viz: 832, at 7, A. M., 721 at 2, P. M., and 853 at 9, P. M.

The amount of rain was large. A fall of rain was usually accompanied by a fall of the barometer, and constantly so when there was a high wind. The prevailing winds during the month were from the west and south-west, but the month generally was very still. From the 18th to the 21st there was at no time anything more than a very light breeze.

